# The potential role of mitochondrial impairment in the pathogenesis of imatinib-induced renal injury

**DOI:** 10.1016/j.heliyon.2019.e01996

**Published:** 2019-06-22

**Authors:** Ehsan Emadi, Narges Abdoli, Vahid Ghanbarinejad, Hamid Reza Mohammadi, Khadijeh Mousavi Mobarakeh, Negar Azarpira, Zahra Mahboubi, Hossein Niknahad, Reza Heidari

**Affiliations:** a Pharmaceutical Sciences Research Center, Shiraz University of Medical Sciences, Shiraz, Iran; b Department of Pharmacology and Toxicology, School of Pharmacy, Shiraz University of Medical Sciences, Shiraz, Iran; c Iran Food and Drug Administration (IFDA), Ministry of Health, Tehran, Iran; d Transplant Research Center, Shiraz University of Medical Sciences, Shiraz, Iran

**Keywords:** Pharmaceutical science, Toxicology, Renal system, Pharmacology, Energy crisis, Oxidative stress, Mitochondria, Electrolyte imbalance, Renal injury, Fanconi syndrome, ATP

## Abstract

Imatinib is a tyrosine kinase inhibitor widely administered against chronic myeloid leukemia. On the other hand, drug-induced kidney proximal tubular injury, electrolytes disturbances, and renal failure is a clinical complication associated with imatinib therapy. There is no precise cellular mechanism(s) for imatinib-induced renal injury. The current investigation aimed to evaluate the role of mitochondrial dysfunction and oxidative stress in the pathogenesis of imatinib nephrotoxicity. Rats received imatinib (50 and 100 mg/kg, oral, 14 consecutive days). Serum and urine biomarkers of renal injury and markers of oxidative stress in the kidney tissue were assessed. Moreover, kidney mitochondria were isolated, and mitochondrial indices, including mitochondrial depolarization, dehydrogenases activity, mitochondrial permeabilization, lipid peroxidation (LPO), mitochondrial glutathione levels, and ATP content were determined. A significant increase in serum (Creatinine; Cr and blood urea nitrogen; BUN) and urine (Glucose, protein, gamma-glutamyl transferase; γ-GT, and alkaline phosphatase; ALP) biomarkers of renal injury, as well as serum electrolytes disturbances (hypokalemia and hypophosphatemia), were evident in imatinib-treated animals. On the other hand, imatinib (100 mg/kg) caused an increase in kidney ROS and LPO. Renal tubular interstitial nephritis, tissue necrosis, and atrophy were evident as tissue histopathological changes in imatinib-treated rats. Mitochondrial parameters were also adversely affected by imatinib administration. These data represent mitochondrial impairment, renal tissue energy crisis, and oxidative stress as possible mechanisms involved in the pathogenesis of imatinib-induced renal injury and serum electrolytes disturbances.

## 1 Introduction

Tyrosine kinase inhibitors are widely administered against chronic myeloid leukemia in human. Although these drugs had a revolutionary effect on the management of malignancies in patients, several adverse reactions have been associated with their clinical use (Cismowski 2007a, 2007b). Imatinib is a tyrosine kinase inhibitor clinically applied against leukemia and metastatic gastrointestinal stromal tumors (Cismowski, 2007a). Nausea, vomiting, abdominal pain, and elevated serum transaminase level are associated with imatinib therapy (Cismowski, 2007a; Francis et al., 2015; Cross et al., 2006; Tonyali et al., 2010). On the other hand, nephrotoxicity and acute renal failure might accompany imatinib administration (Cismowski, 2007a). There is no precise mechanism for imatinib-induced renal injury.

It has been found that several xenobiotics, including many pharmaceuticals, can induce chemical transport defect in the proximal renal tubules (Hall et al., 2014; Izzedine et al., 2003; Kintzel, 2001; Heidari, 2019). This complication leads to serum electrolytes disturbances and the abnormal level of chemicals such as glucose, proteins, and amino acids in the urine (Hall et al., 2014; Heidari, 2019). Although the clinical feature of xenobiotics-induced renal injury and electrolytes disturbances is well-described, the mechanism(s) underlying this complication remained obscure. Several cases of electrolytes abnormalities have been reported in imatinib-treated patients (Mulder et al., 2012; François et al., 2008; Berman et al., 2006). Serum electrolytes abnormality, including hypophosphatemia and hypokalemia, are well-described events associated with imatinib therapy (Mulder et al., 2012; Berman et al., 2006).

Chemicals reabsorption process in the kidney tubule is an active and energy (ATP)-dependent process (Shaik et al., 2008; Soltoff and Mandel, 1984). Renal proximal tubule contains numerous mitochondria which guarantee enough ATP level for the chemicals reabsorption process (Shaik et al., 2008; Soltoff and Mandel, 1984). The electrochemical sodium gradient created by the Na^+^/K^+^ ATPase pump is used for the reabsorption process of many chemicals by proximal renal tubule (Lote, 2012). Na^+^/K^+^ ATPase activity is an energy (ATP) dependent process. In the kidneys, the driving force provided by the Na^+^ export is used for importing several chemicals such as glucose, vitamins, phosphate, low molecular proteins, amino acids, and some other organic compounds into the proximal tubule cells, and subsequently to the bloodstream (Chakraborti et al., 2016). Hence, cellular mitochondria are potential targets for xenobiotics-induced cytotoxicity (Niknahad et al., 2017). Any defect in the proper mitochondrial function might interfere with the chemicals reabsorption process in the kidney.

The current investigation was designed to evaluate the effect of imatinib on renal tissue mitochondrial function as a potential cellular target of xenobiotics cytotoxicity. The data obtained from the current study might help to develop new therapeutic/preventive strategies against imatinib nephrotoxicity in clinical settings.

## 2 Materials and methods

### 2.1 Chemicals

2′,7′-Dichlorofluorescein diacetate (DCFH-DA), trichloroacetic acid (TCA), 3-[4,5dimethylthiazol-2-yl]-2,5-diphenyltetrazolium bromide (MTT), sucrose, perchloric acid, D-mannitol, imatinib mesylate, Rhodamine123 (Rh 123), 3-(N-morpholino) propane sulfonic acid (MOPS), 2, 4-dinitrofluorobenzene (DNFB), sodium acetate, acetic acid glacial, 2, 4, 6-tripyridyl-s-triazine (TPTZ), iodoacetic acid, dithiothreitol (DTT), 6-hydroxy-2,5,7,8-tetramethylchroman-2-carboxylic acid (Trolox), and thiobarbituric acid (TBA) were purchased from Sigma (Sigma-Aldrich, St. Louis, MO). Kits for evaluating serum biomarkers of renal injury were obtained from Pars Azmun^®^ (Tehran, Iran). Ethylenediaminetetraacetic acid (EDTA), reduced glutathione (GSH), malondialdehyde (MDA), fatty acid-free bovine serum albumin (BSA) fraction V, oxidized glutathione (GSSG), ferric chloride hexahydrate (FeCl_3_.6H_2_O), coomassie brilliant blue, 4-(2-hydroxyethyl)-1-piperazineethanesulfonic acid (HEPES), meta-phosphoric acid, n-butanol, and 2-Amino-2-hydroxymethyl-propane-1,3-diol-hydrochloride (Tris-HCl) were obtained from Merck (Merck KGaA, Darmstadt, Germany). All salts used for preparing buffer solutions were of the analytical grade and obtained from Merck (Merck KGaA, Darmstadt, Germany).

### 2.2 Animals

Male Sprague-Dawley rats (n = 24) weighing 200–250 g were obtained from the Laboratory Animal Breeding Center, Shiraz University of Medical Sciences (Shiraz, Iran). Animals were housed in an ambient temperature of 23±1 °C with ≈40% of relative humidity. Rats had free access to a standard pellet chow (Behparvar^®^, Tehran, Iran) and tap water. All procedures involving animal use were under the guidelines for care and use of laboratory animals and were endorsed by the institutional ethics committee at Shiraz University of Medical Sciences, Shiraz, Iran (95-01-36-12284).

### 2.3 Experimental setup

Animals were randomly allotted into three groups (n = 8 in each group). Rats were treated as follows: **1**) Control (Vehicle-treated group), **2**) Imatinib (50 mg/kg/day, oral); **3**) Imatinib (100 mg/kg/day, oral). Normal saline was used as imatinib vehicle (2.5 mL/kg). The imatinib dose and time of administration (14 days) were selected based on previous studies (Herman et al., 2011).

### 2.4 Specimen collection and serum and urine biochemistry

At the end of experiments (At day 15^th^, 24 hours after the final dose of imatinib) (Herman et al., 2011), urine was collected after the micturition during animal handling (100 μL). Samples were diluted with cold saline (NaCl 0.9% w:v, 4 °C) and centrifuged (1000 *g*, 5 min, 8 °C). The clear supernatant was used for urinalysis. Animals were anesthetized (ketamine/xylazine; 100/10 mg/kg, i.p) and their blood and kidney samples were collected. Blood was collected from the abdominal aorta, transferred to standard tubes (Improvacuter^®^; gel and clot activator-coated tubes; Guangzhou, China) and centrifuged (3000 *g*, 10 minutes, 8 °C) to prepare serum. An autoanalyzer (Mindray BS-200^®^, China) and standard kits (Pars Azmun^®^, Tehran, Iran) were employed to assess serum creatinine (Cr), phosphate, uric acid, calcium, blood urea nitrogen (BUN), glucose, gamma-glutamyl transpeptidase (γ-GT), alkaline phosphatase (ALP), and protein (Heidari et al., 2016; Jamshidzadeh et al., 2015). Sodium (Na^+^) and potassium (K^+^) levels were measured using a flame photometer (Clinical Flame Photometer, Sherwood Scientific, UK).

### 2.5 Histopathological assessment

For histopathological assessments of the kidney tissue, samples were fixed in a buffered formalin solution (10% formaldehyde in distilled water, 0.4% sodium phosphate monobasic, NaH_2_PO_4_, 0.64% sodium phosphate dibasic, Na_2_HPO_4_, and; pH = 7.4). Paraffin-embedded sections (5 μm) of renal tissue were stained with hematoxylin and eosin (H&E) (Moezi et al., 2013). Renal histopathological alterations were scored as previously described based on a model of toxic nephropathy (Zheng et al., 2005). The Masson trichrome staining was applied to assess kidney tissue fibrosis (Heidari et al., 2019b). Samples were analyzed in a blind fashion (Olympus CX21^®^, Light microscope, Japan).

### 2.6 Kidney tissue reactive oxygen species (ROS)

Samples of the kidney tissue (1:10 w: v) were homogenized in ice-cooled Tris-HCl buffer (40 mM, pH = 7.4). Then, 100 μL of tissue homogenate was mixed with 1 mL of Tris-HCl buffer (40 mM, pH = 7.4) and 10 μL of 2′, 7′-dichlorofluorescein diacetate (Final concentration 10 μM). Samples were incubated in the dark (15 min, 37 °C in a shaker incubator). Finally, the fluorescence intensity (FI) of the samples was assessed using a FLUOstar Omega^®^ multifunctional microplate reader (BMG Labtech Inc., Germany, λ _excitation_ = 485 nm and λ _emission_ = 525 nm) (Socci et al., 1999; Jamshidzadeh et al., 2015; Ahmadian et al., 2018; Eftekhari et al., 2018).

### 2.7 Kidney and mitochondria glutathione content

The reduced (GSH) and oxidized (GSSG) glutathione levels in the kidney tissue and isolated mitochondria were measured by an HPLC method (Meeks and Harrison, 1991). The technique is based on the formation of S-carboxymethyl derivatives of free thiols with iodoacetic acid followed by transformation of free amino groups to 2,4-dinitrophenyl derivatives by reaction with fluoro-2,4-dinitrobenzene. Deproteinized samples (TCA 50%) were derivatized with iodoacetic acid and fluoro-2,4-dinitrobenzene and analyzed using an NH_2_ column (250 mm × 4 mm ID, Bischoff chromatography, Leonberg, Germany) flow rate 1 mL/min, and the UV detector (λ = 254 nm) (Meeks and Harrison, 1991). The mobile phases consisted of buffer A (Water: Methanol; 1:4 v/v) and buffer B (Buffer A: Acetate buffer; 4:1 v/v) and a gradient method with a steady increase of buffer B to 95% (in 20 min) (Meeks and Harrison, 1991). GSH and GSSG were used as external standards. Kidney tissue (200 mg) were homogenized in 5 mL of Tris-HCl buffer (40 mM; pH = 7.4; 4 °C). Then, 500 μL of trichloroacetic acid (TCA, 50% w: v, 4 °C) was added to 2 mL of the tissue homogenate. Mitochondria samples (500 μL; 10 mg protein/mL) were also treated with 50 μL of TCA (50% w: v). Afterward, samples were mixed well and incubated on ice (10 min). Afterward, samples were centrifuged (15,000 g, 15 min, 4 °C) and 1 mL of the supernatant was collected, and the NaOH: NaHCO_3_ (2 M: 2 M) was added (≈300 μL) till the gas production was subsided. Afterward, 100 μL of iodoacetic acid (1.5% w: v in water) was added, and samples were incubated in the dark (1 h, 4 °C). After the incubation period, 500 μL of 2, 4-dinitrofluorobenzene (DNFB; 1.5% w: v in absolute ethanol) was added and incubated in the dark (25 °C, at least for 24 hours). Finally, 25 μL of samples were injected into the previously described HPLC system (Truong et al., 2006; Meeks and Harrison, 1991).

### 2.8 Lipid peroxidation

Thiobarbituric acid reactive substances (TBARs) were measured as an index of lipid peroxidation in the kidney tissue (da Silva et al., 2012; Yao et al., 2009). Briefly, 1 mL of kidney tissue homogenate (10% w/v in Tris-HCl buffer, 40 mM, pH = 7.4, 4 °C) was added to 3 mL of a reaction mixture consisted of trichloroacetic acid (15 %; w/v), thiobarbituric acid (0.375%, w/v), and 1 mL of hydrochloric acid (0.2 N) (pH = 2). Samples were mixed well and heated in a water bath (100 °C, 45 minutes). Finally, 2 mL of n-butanol was added and vigorously mixed. Samples were centrifuged (10000 g for 10 minutes) and the absorbance of developed color in n-butanol phase was measured (λ = 532 nm, EPOCH plate reader, BioTek^®^ Instruments, Highland Park, USA) (Heidari et al., 2017; Ommati et al., 2019).

### 2.9 Ferric reducing antioxidant power (FRAP) of kidney tissue

The total antioxidant capacity (TAC) of the kidney tissue was measured with the FRAP assay (Gülçin, 2015). FRAP assay measures the formation of Fe^2+^-tripyridyltriazine compound (blue colored) from the oxidized Fe^3+^ (colorless) form by the action of electron-donating antioxidants (Katalinic et al., 2005; Gülçin, 2015; Heidari et al., 2017). The working FRAP reagent consisted of freshly prepared 10 volumes of acetate buffer (300 mmol/L, pH = 3.6), 1 volume of ferric chloride (20 mmol/L), and 1 volume of TPTZ (10 mmol/L dissolved in HCl). Kidney tissue was homogenized in an ice-cooled (4 °C) 40 mM Tris buffer Tris-HCl (pH = 7.4) containing 200 mM sucrose and 5 mM DTT (Heidari et al., 2018a). Then, 50 μL of tissue homogenate and 150 μL of deionized water was added to 1.5 mL of the FRAP reagent. Samples were incubated at 37 °C (5 min, in the dark). Finally, the absorbance of developed color was measured (λ = 595 nm, EPOCH plate reader, BioTek^®^ Instruments, Highland Park, USA) (Alía et al., 2003; Heidari et al., 2019c).

### 2.10 Kidney mitochondria isolation

Cellular mitochondria were isolated by differential centrifugation of the kidney tissue homogenate (Fernández-Vizarra et al., 2010). Briefly, rat kidney was washed and minced in an ice-cold (4 °C) buffer medium containing 70 mM mannitol, 2 mM HEPES, 0.5 mM EGTA, 220 mM sucrose, and 0.1% BSA, pH = 7.4). Minced tissue was transported into the new buffer (5 ml buffer/1g of the kidney) and homogenized. First, unbroken cells and nuclei were pelleted (1000 *g*, 10 min, 4 °C); second, the supernatant was centrifuged (10,000 *g*, 10 min, 4 °C) to pellet the mitochondria fraction (Dark brown). The current step was repeated at least three times using a fresh buffer medium to increase the mitochondrial yield (Fernández-Vizarra et al., 2010). Finally, the mitochondrial pellet was suspended in a buffer containing 70 mM mannitol, 220 mM sucrose, 2 mM HEPES, and 0.5 mM EGTA, pH = 7.4, except for the mitochondria used to assess mitochondrial depolarization and mitochondrial swelling, which were suspended in 68 mM mannitol, 10 mM KCl, 220 mM sucrose, 5 mM KH_2_PO_4_, 50 μM EGTA, 2 mM MgCl_2_ and 10 mM HEPES, pH = 7.2, and swelling buffer (65 mM KCl, 125 mM sucrose, 10 mM HEPES, pH = 7.2) (Caro et al., 2012). Samples protein concentrations were measured based on the Bradford method to standardize the obtained data (Bradford, 1976).

### 2.11 Mitochondrial dehydrogenases activity

A colorimetric method based on the 3-(4, 5-dimethylthiazol-2-yl)-2,5-diphenyltetrazolium bromide assay was applied for the determination of mitochondrial dehydrogenases activity (Mosmann, 1983; Niknahad et al., 2015; Heidari and Niknahad, 2019). Briefly, a mitochondrial suspension (1 mg protein/mL) was incubated with MTT (40 μL of 0.4% w: v solution) for 30 min (37 °C in the dark). The product of purple formazan crystals was dissolved in dimethyl sulfoxide (DMSO, 1 mL) and the optical density (OD) was measured (λ = 570 nm, EPOCH plate reader, Bio-Tek^®^ Instruments, Highland Park, USA) (Heidari and Niknahad, 2019; Ommati et al., 2018).

### 2.12 Mitochondrial depolarization

Mitochondrial capability to capture the cationic fluorescent dye, rhodamine 123, was used a method to estimate mitochondrial depolarization (Heidari et al. 2012, 2013; Caro et al., 2012; Heidari and Niknahad, 2019). Rhodamine 123, accumulates in intact mitochondria by facilitated diffusion. When the mitochondrion is damaged and depolarized, there is no facilitated diffusion, and the amount of rhodamine 123 in the supernatant is increased (Heidari et al. 2012, 2013; Caro et al., 2012; Heidari and Niknahad, 2019; Ahmadian et al., 2016). In the current study, the mitochondrial fractions (1 mg protein/mL) were incubated with 10 μL of rhodamine 123 (Final concentration 10 μM) in the mitochondrial depolarization assay buffer (30 min, 37 °C, in the dark). Afterward, samples were centrifuged (15000 g, 5 min, 4 °C) and the fluorescence intensity of the supernatant was monitored (FLUOstar Omega^®^ multifunctional microplate reader, BMG Labtech Inc., Germany, λ _excitation_ = 485 nm and λ _emission_ = 525 nm) (Caro et al., 2012; Niknahad et al., 2016).

### 2.13 Mitochondrial permeabilization and swelling

Analysis of mitochondrial permeabilization was estimated through the alterations in light absorbance as monitored spectrophotometrically (λ = 540 nm, 30 °C, EPOCH plate reader, Bio-Tek^®^ Instruments, Highland Park, USA) (Caro et al., 2012). Kidney mitochondria samples (0.5 mg protein/mL) were suspended in swelling buffer, and the absorbance was monitored at λ = 540 nm during 30 minutes of incubation. A decrease in absorbance indicates an increase in mitochondrial swelling (Caro et al., 2012).

### 2.14 Lipid peroxidation in isolated kidney mitochondria

Thiobarbituric acid-reactive substances (TBARS) test was used to assess lipid peroxidation in isolated kidney mitochondria (Caro et al., 2012). As previous studies reported, sucrose interferes with the TBARS test (Caro et al., 2012). Hence, isolated mitochondria were washed once (to remove sucrose) in ice-cooled MOPS-KCl buffer (100 mM KCl, 50 mM MOPS, pH = 7.4, 4 °C), and re-suspended in MOPS–KCl buffer (Caro et al., 2012). Afterward, the mitochondrial suspension (1 mL, 1 mg protein/mL) was added with twice its volume of a mixture containing thiobarbituric acid (0.375% w: v), trichloroacetic acid (15% w: v), 1 mL HCl (0.2 N), and Trolox (0.5 mM). Samples were heated in a water bath (15 min, 100 °C) (Caro et al., 2012). After centrifugation (15000 *g*, 10 minutes), the absorbance of the supernatant was measured (λ = 532 nm, EPOCH plate reader; BioTek^®^ Instruments, Highland Park, USA) (Caro et al., 2012).

### 2.15 Mitochondrial ATP level

A luciferase-luciferin-based kit (Enliten^®^ from Promega, Madison, USA) was used to assess mitochondrial ATP content (Heidari et al., 2018b; Ommati et al., 2018). Samples and buffer solutions were prepared based on the kit instructions. Briefly, mitochondrial samples (500 μL, 1 mg protein/mL) were treated with 200 μL of ice-cooled TCA solution (0.3% w: v in MiliQ water, 4 °C) and centrifuged (15,000 *g*, 15 min, 4 °C). Afterward, 100 μL of the supernatant was treated with 100 μL of ATP kit content in the dark, and the luminescence intensity of samples was measured (λ = 560 nm, FLUOstar Omega^®^ multifunctional microplate reader, BMG Labtech Inc., Ortenberg, Germany) (Heidari et al., 2018b; Ommati et al., 2018).

### 2.16 Statistical analysis

Data are given as the mean ± SD. The one-way analysis of variance (ANOVA) with Tukey's multiple comparisons as the *post hoc* test was used for data comparison. P < 0.05 was considered a statistically significant difference.

## 3 Results

Imatinib-treated animals developed biochemical evidence of renal injury and serum electrolytes disturbances (Table 1). Elevated serum Cr, and BUN along with hypokalemia, and hypophosphatemia were detected in imatinib-treated (100 mg/kg) animals (Table 1). Furthermore, a significant increase in urine protein, glucose, γ-GT, and ALP was evident in the imatinib group (100 mg/kg) (Table 2). Several biochemical parameters were significantly higher in imatinib 100 mg/kg treated group in comparison with 50 mg/kg of imatinib (Table 1).

**Table 1:** Serum biochemical assessment in imatinib-treated rats.

Parameters assessed	Control	Imatinib 50 mg/kg	Imatinib 100 mg/kg
Glucose (mg/dl)	115 ± 9	110 ± 8	88 ± 5*^,a^
K^+^ (mmol/l)	5.8 ± 0.9	3.6 ± 0.3*	3.5 ± 0.6*^,a^
Phosphate (mg/dl)	3.5 ± 0.12	2.4 ± 0.4*	2.1 ± 0.2*
Ca^2+^ (mg/dl)	4.9 ± 0.5	4 ± 0.5	4.8 ± 0.5
Na^+^ (mmol/l)	91 ± 5	81 ± 6	62 ± 4*^,a^
Uric acid (mg/dl)	1.9 ± 0.3	1.5 ± 0.3	0.8 ± 0.2*
Total protein (mg/dl)	7.2 ± 0.2	6.8 ± 0.3	6.8 ± 0.5
Blood Urea Nitrogen (mg/dl)	44 ± 3	43 ± 6	60 ± 4*
Creatinine (mg/dl)	0.28 ± 0.04	0.33 ± 0.04	0.59 ± 0.08*^,a^

Data are given as mean ± SD (n = 8).

*Indicates significantly different as compared with the control group (P < 0.001).

^a^ Indicates significantly different as compared with imatinib 50 mg/kg group (P < 0.05).

**Table 2:** Urine biochemistry of imatinib-treated animals.

	Control	Imatinib 50 mg/kg	Imatinib 100 mg/kg
Total protein (mg/dl)	0.46 ± 0.1	0.73 ± 0.2	1.3 ± 0.2*^,a^
γ-GT (U/l)	2009 ± 353	2808 ± 305	3420 ± 286*
Glucose (mg/dl)	74 ± 5	93 ± 10	119 ± 11*
Alkaline Phosphatase (U/l)	2054 ± 241	2129 ± 254	2909 ± 169*

Data are given as mean ± SD (n = 8).

*Indicates significantly different as compared with the control group (P < 0.001).

^a^ Indicates significantly different as compared with imatinib 50 mg/kg group (P < 0.05).

**Fig. 1:**
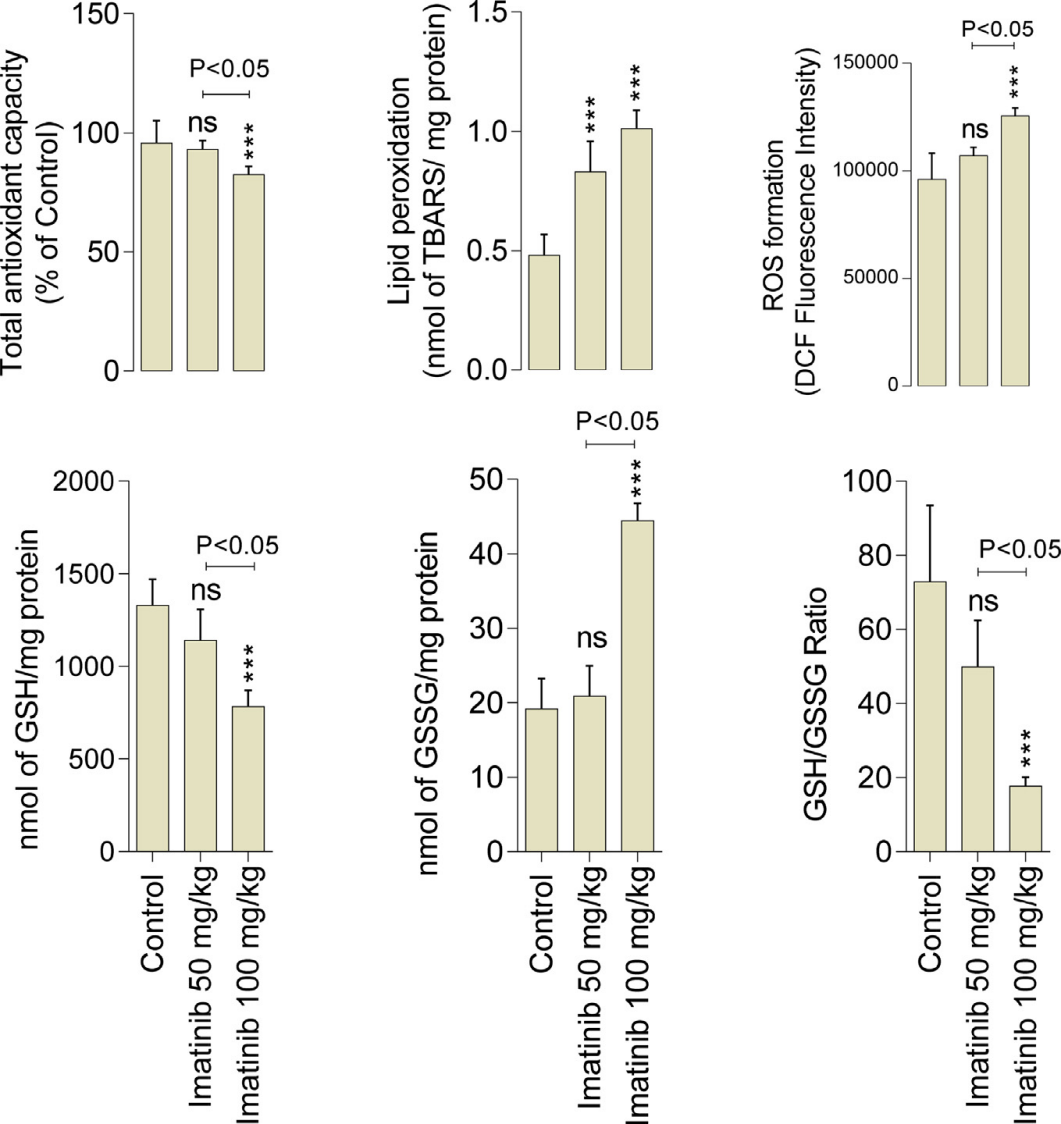
Markers of oxidative stress in the kidney tissue of imatinib-treated rats. ROS: Reactive Oxygen Species, DCF: Dichlorofluorescein, GSH: Glutathione, GSSG: Oxidized glutathione. Data are represented as mean ± SD (n = 8). Asterisks indicate significantly different as compared with control group (*P < 0.05; ***P < 0.001). ns: not significant as compared with the control group.

A significant increase in the level of oxidative stress biomarkers of the kidney was evident in imatinib-treated animals (Fig. 1). It was found that imatinib (100 mg/kg) caused an increase in the kidney ROS level, GSSG, and lipid peroxidation (Fig. 1). Moreover, renal tissue antioxidant capacity was hampered, and glutathione (GSH) reservoirs were depleted in imatinib-treated rats (Fig. 1). The effects of imatinib on kidney oxidative stress biomarkers was dose-dependent (Fig. 1).

**Fig. 2:**
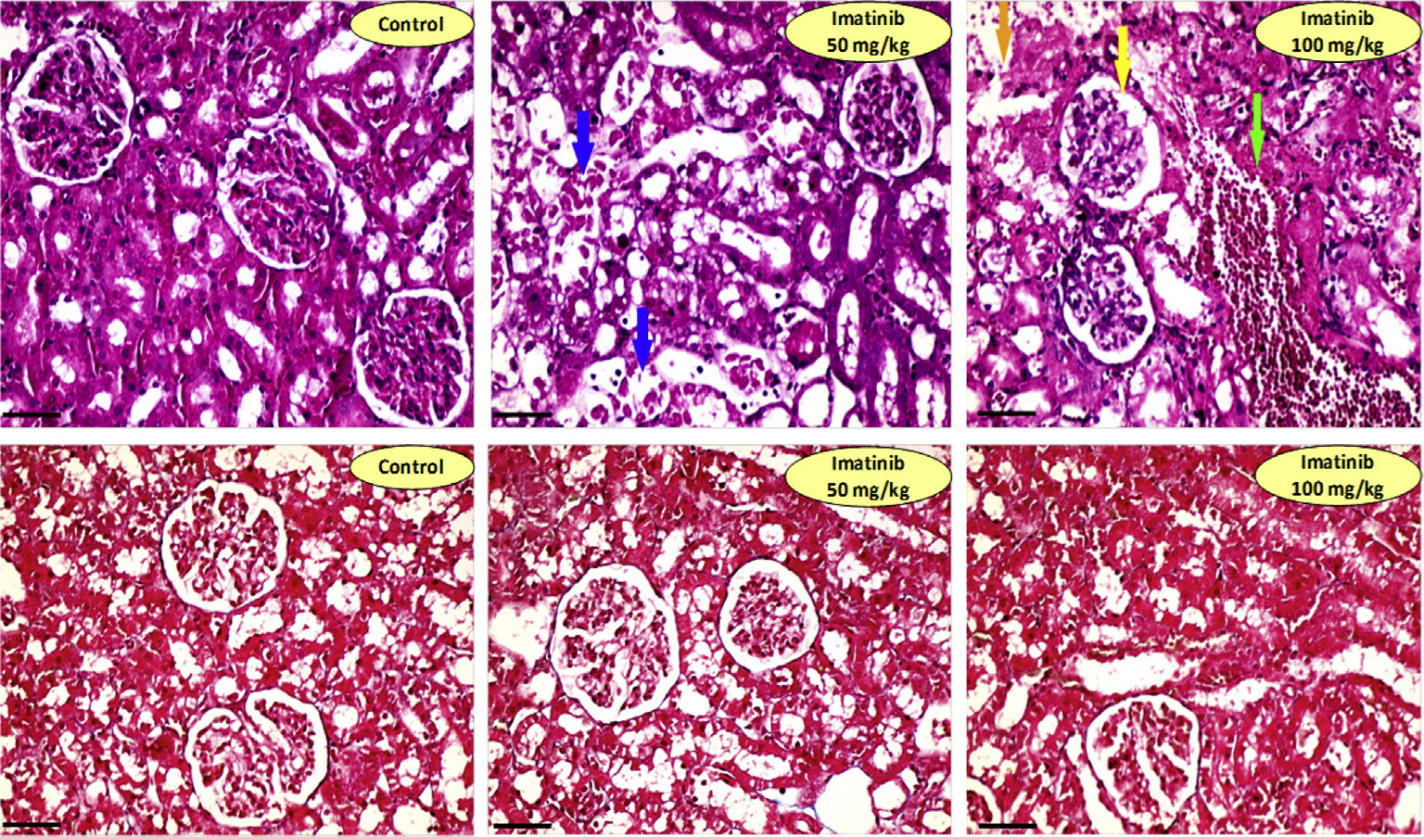
Kidney tissue histopathological alterations in imatinib-treated rats. Top row: H&E staining. Signs of moderate (++) tissue necrosis (Orange arrow), severe (+++) glomerular dilation (Blue arrow), mild (+) tubular degeneration (Yellow arrow), and mild (+) vascular congestion (Green arrow) were detected in imatinib 100 mg/kg-treated animals. Only mild (+) glomerular atrophy was evident in imatinib 50 mg/kg group. No sign of kidney tissue fibrosis was detected in imatinib-treated animals (Lower row; Masson trichrome staining). Magnification: ×400. Scale bars: 50 μm.

**Fig. 3:**
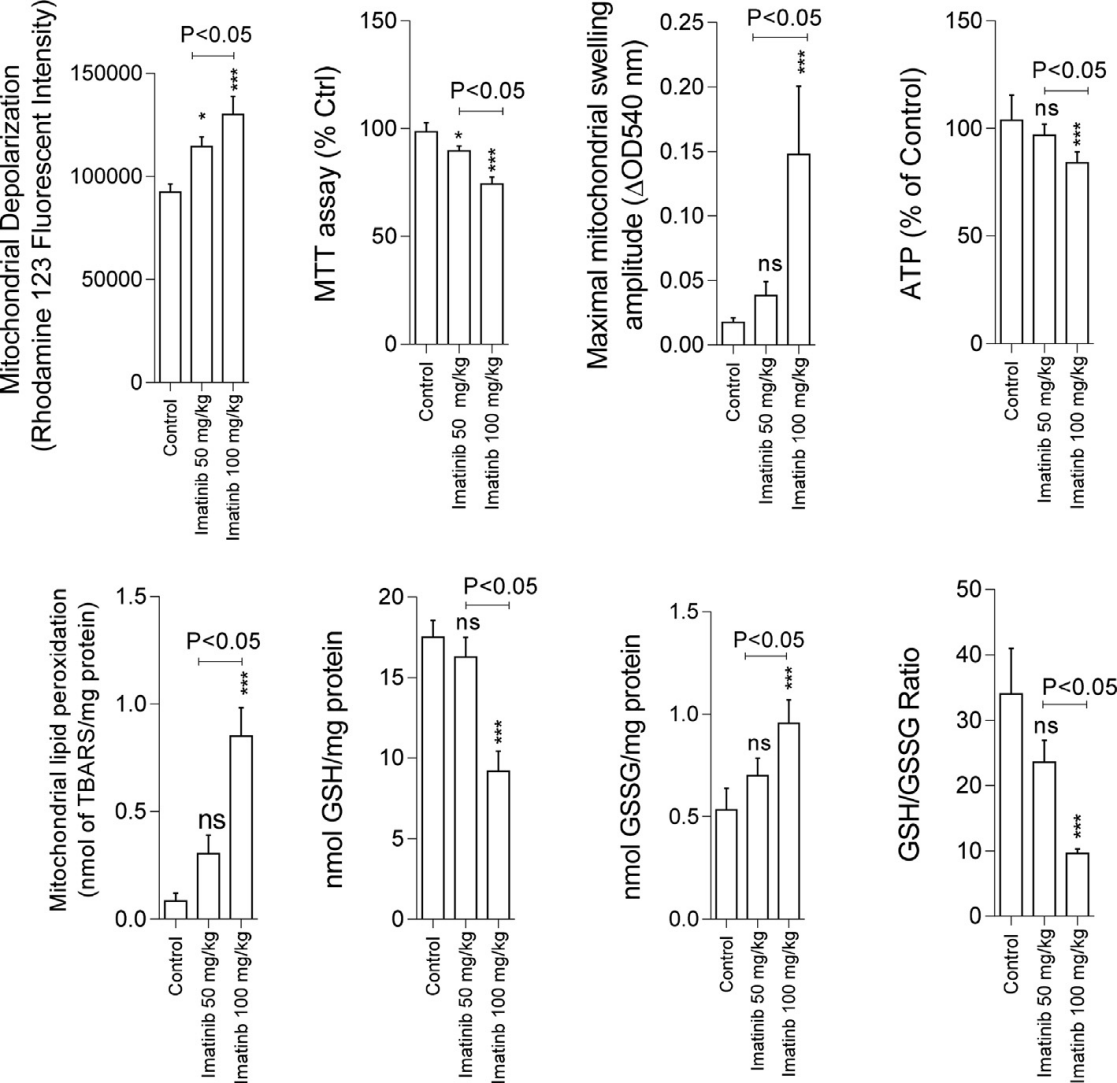
Deterioration in mitochondrial indices of functionality in the kidney tissue of imatinib-treated rats. ROS: Reactive Oxygen Species, DCF: Dichlorofluorescein, GSH: Glutathione, GSSG: Oxidized glutathione, ATP: Adenosine triphosphate, TBARS: Thiobarbituric acid reactive substances, MTT: Methyl tetrazolium. Data are given as mean ± SD (n = 8). *** Indicates significantly different as compared with the control group (P < 0.001). ns: not significant as compared with the control group.

Histopathological changes of renal tissue in imatinib-treated (100 mg/kg) rats were included interstitial nephritis, tissue necrosis, and atrophy (Fig. 2). Mild interstitial nephritis was also detected in imatinib (50 mg/kg)-treated animals (Fig. 3 and Table 2). No sign of kidney tissue fibrosis was detected in imatinib-treated rats (Masson trichrome staining) when it was compared with the control (vehicle-treated) group (Fig. 2).

A marked decrease in dehydrogenases activity was detected in the kidney mitochondria isolated from imatinib-treated (100 mg/kg) animals (Fig. 3). Further assessment of kidney mitochondria derived from the imatinib-treated group (100 mg/kg) revealed a significant increase in mitochondrial permeabilization and mitochondrial depolarization (Fig. 3). It was found that mitochondrial glutathione stores and ATP levels were decreased in imatinib-treated animals (Fig. 3). Imatinib administration (100 mg/kg) also increased mitochondrial lipid peroxidation and GSSG content (Fig. 3). The adverse effects of imatinib on kidney mitochondria indices was dose-dependent (Fig. 3).

## 4 Discussion

Imatinib is clinically administered against chronic myeloid leukemia and metastatic gastrointestinal stromal tumors (Cismowski 2007a, 2007b). On the other hand, renal injury and electrolytes abnormalities are associated with imatinib therapy (Mauro et al., 2012; Lipton et al., 2011; Liamis et al., 2016). There is no clear mechanism for imatinib-induced renal injury. The data obtained from the current investigation mention the potential role of mitochondrial injury and oxidative stress in the pathogenesis of imatinib-induced nephrotoxicity and serum electrolytes imbalance.

Renal reabsorption defect and serum electrolytes abnormalities are associated with a wide range of xenobiotics, including many pharmaceuticals (Heidari, 2019; Heidari et al., 2019a; Izzedine et al., 2003). Several human cases of electrolytes waste have been reported in imatinib-treated patients (Osorio et al., 2007; François et al., 2008; Kantarjian et al., 2012). Hypophosphatemia is a common electrolyte abnormality accompanied the administration of tyrosine kinase inhibitor drugs (Osorio et al., 2007; François et al., 2008; Kantarjian et al., 2012). Physiologically, the reabsorption process of many chemicals takes place in the proximal renal tubule in an energy-dependent manner (Lote, 2012). In this context, the Na^+^/K^+^ ATPase pump plays a crucial role in the chemicals reabsorption process (Lote, 2012). Na^+^/K^+^ ATPase consumes ATP to produce a chemical gradient of Na^+^ ion, which finally is used for the transport of chemicals to the bloodstream (Chakraborti et al., 2016; Lote, 2012). High and constant dependence of proximal tubular reabsorption process to the ATP, highlights the importance of proper mitochondrial function and energy production. In the current study, we found that imatinib administration significantly decreased mitochondria ATP level in the kidney (Fig. 3). It was also found that mitochondrial membrane potential was dissipated in the renal mitochondria isolated from imatinib-treated animals (Fig. 3). Hence, interference with renal mitochondrial function seems to be a potential mechanism for imatinib cytotoxicity, renal injury, and defect in renal reabsorption process. In the current study, we found signs of hypophosphatemia and hypokalemia in imatinib-treated rats (Table 1). On the other hand, significant proteinuria and glucosuria were detected in imatinib-exposed animals (Table 1). Therefore, the occurrence of disturbances in the chemicals reabsorption process is possibly due to drug-induced mitochondrial impairment, the lack of adequate ATP, and inappropriate Na^+^/K^+^ ATPase pump function in the renal proximal tubule. Moreover, xenobiotics-induced disturbances in cellular energy metabolism might deteriorate the reabsorption of chemicals in segments beyond the proximal tubule (Palmer and Schnermann, 2015; Chen et al., 2016). All these data mention that drug-induced mitochondrial impairment and cellular energy crisis play a pivotal role in renal dysfunction and serum electrolytes disturbances (Heidari, 2019).

Oxidative stress plays a critical role in the mechanism of xenobiotics cytotoxicity (Quiros et al., 2011; Tafazoli et al., 2005). In the current study, it was found that imatinib administration to rats was associated with oxidative stress in the kidney tissue (Fig. 1). Tissue antioxidant capacity was decreased while ROS formation and lipid peroxidation were increased in imatinib-treated (100 mg/kg) animals (Fig. 1). Lower GSH level and GSH/GSSG ratio, which is a good characterization for the occurrence of oxidative stress in biological systems, was evident in imatinib-treated (100 mg/kg) group (Fig. 1). On the other hand, mitochondrial injury and oxidative stress are two mechanistically interconnected events (Brookes et al., 2004). Severe oxidative stress adversely affects mitochondrial function (Brookes et al., 2004). Cellular mitochondria are also the primary site of intracellular ROS production (Brookes et al., 2004). Hence, imatinib-induced mitochondrial dysfunction could hasten oxidative stress in the kidney tissue and vice versa. All these data mention the importance of oxidative stress and its associated events in the mechanism of imatinib-induced renal injury.

Cardiotoxicity is a severe adverse effect associated with tyrosine kinase inhibitors, including imatinib (Schmidinger et al., 2008; Chu et al., 2007; Kerkelä et al., 2006). Several mechanisms have been proposed for imatinib-induced cardiotoxicity (Chu et al., 2007; Will et al., 2008). Interestingly, it has been found that imatinib-induced mitochondrial dysfunction might be involved in the mechanism of drug cardiotoxicity (Chu et al., 2007; Will et al., 2008). Mitochondrial swelling and abnormal mitochondrial configurations have been documented in electron microscope images of tyrosine kinase-exposed cardiomyocytes (Chu et al., 2007). These data mention cellular mitochondria as a potential target for imatinib cytotoxicity. In the current investigation, we found that kidney mitochondria might also be a potential target for imatinib to induce kidney injury and serum electrolytes disturbances.

Previous investigations also mentioned 50 mg/kg of imatinib for 14 consecutive days as the toxic doses of this drug in other organs (*e.g.*, Heart tissue) (Herman et al., 2011). In the current study, it was found that 50 mg/kg of imatinib was able to change some renal injury and serum electrolytes abnormality markers (Tables 1 and 2). However, it seems that the higher dose of imatinib (100 mg/kg) was more toxic toward the kidney tissue. Previous studies mentioned renal fibrosis as a typical feature of drug-induced renal tubule injury and Fanconi syndrome (Hall et al., 2014). Moreover, some studies revealed renal tissue fibrotic alterations upon tyrosine kinase drug therapy (Pou et al., 2003). However, some other studies reported no sign of renal fibrosis in human cases of imatinib-induced renal failure (Foringer et al., 2005). In the current study, we found no sign of kidney tissue fibrosis in imatinib-treated (50 and 100 mg/kg) animals. Different factors, including drug dose and the duration of study, might be involved in the development of renal tissue fibrosis associated with imatinib administration.

Current data could provide insights to determine the mechanisms responsible for imatinib-induced renal injury. Further investigations are warranted to reveal the clinical significance of these data and developing therapeutic/preventive strategies against imatinib-induced renal injury and serum electrolytes disturbances.

## Declarations

### Author contribution statement

Reza Heidari, Hossein Niknahad: Conceived and designed the experiments; Performed the experiments; Analyzed and interpreted the data; Contributed reagents, materials, analysis tools or data; Wrote the paper.

Ehsan Emadi, Narges Abdoli, Vahid Ghanabrinejad, Hamid Reza Mohammadi, Khadijeh Mousavi Mobarakeh, Negar Azarpira, Zahra Mahboubi: Performed the experiments; Analyzed and interpreted the data; Contributed reagents, materials, analysis tools or data; Wrote the paper.

### Funding statement

This work was supported by the Vice Chancellor of Research Affairs of Shiraz University of Medical Sciences (Grant number: 95-01-36-12284).

### Competing interest statement

The authors declare no conflict of interest.

### Additional information

No additional information is available for this paper.
